# Severe Late-Onset Drug-Induced Immune Thrombocytopenia Following IFN *β*-1a Treatment: A Case Report of a 52-Year-Old Woman with Relapse-Remitting Multiple Sclerosis

**DOI:** 10.1155/2022/2767031

**Published:** 2022-11-24

**Authors:** Christophe Cisarovsky, Marie Théaudin, Pierre-Alexandre Bart, Grégoire Stalder, Lorenzo Alberio

**Affiliations:** ^1^Division of Internal Medicine, Department of Medicine, Lausanne University Hospital and University of Lausanne, Rue du Bugnon 46, CH-1011 Lausanne, Switzerland; ^2^Division of Neurology, Department of Clinical Neurosciences, Lausanne University Hospital and University of Lausanne, Rue du Bugnon 46, CH-1011 Lausanne, Switzerland; ^3^Service and Central Laboratory of Hematology, Department of Oncology and Department, Laboratories and Pathology, Lausanne University Hospital and University of Lausanne, Rue du Bugnon 46, CH-1011 Lausanne, Switzerland

## Abstract

Interferon *β*-1a (IFN*β*1a) is considered safe in relapsing-remitting multiple sclerosis (RRMS). Drug-induced thrombocytopenia (DITP) is a rare but underreported adverse event that is often confused with other causes of thrombocytopenia. We report the case of a 52-year-old woman who developed limb and oral mucosa petechiae and hematochezia, 10 years after beginning IFN*β*1a. Blood work showed an isolated severe thrombocytopenia and ruled out other autoimmune diseases, viral infections, intravascular hemolysis, and renal impairment. Oral corticosteroids and tranexamic acid were initiated with a favorable platelet response. IFN*β*1a was resumed, leading to recurrence of thrombocytopenia. Platelets came back to normal after intravenous immunoglobulins and IFN*β*1a was definitively discontinued. To our knowledge, this is the first case of drug-induced immune thrombocytopenia (DITP) associated with IFN*β*1a.

## 1. Introduction

Interferons (IFN) *β*-1a and 1b were the first effective treatment for relapsing-remitting multiple sclerosis (RRMS) and are still used as first-line therapies [1]. They are less efficient than most of the recent disease-modifying drugs but have the advantage of an excellent safety profile. However, around 5% of the patients experience hematologic toxicity including mild anemia or thrombocytopenia, which rarely requires dose adjustment or treatment discontinuation [2]. Suggested mechanisms are decreased megakaryocytes proliferation and maturation, and impaired production of platelets [3, 4]. Thrombocytopenia <75 G/L is very uncommon and should prompt further investigations.

Drug-induced thrombocytopenia (DITP) is often not initially recognized and has an estimated incidence rate of 1 case per 100 000 inhabitants per year [5]. This incidence might be substantially higher in hospitalized patients, elderly persons, or those on specific medications. IFN*β* are not listed in the comprehensive database of all substances that can cause thrombocytopenia (http://www.ouhsc.edu/platelets), and immune thrombocytopenia (ITP) is not a side effect that is described by the European Medicines Agency [6]. Of note, cases of thrombotic microangiopathies (hemolytic uremic syndrome and thrombotic thrombocytopenic purpura), which associate thrombocytopenia with anemia and multiorgan failure, have been previously described and are treated differently [7]. Very recently, a further case of thrombotic thrombocytopenic purpura in a woman with relapse-remitting multiple sclerosis treated with IFN*β*-1a was published [8].

Here, we report the first case to our knowledge of a patient with severe relapsing thrombocytopenia following repetitive IFN*β*-1a (Avonex®, Biogen, Switzerland AG) treatment and successfully managed with tranexamic acid, oral corticosteroids, intravenous immunoglobulins, and definitive drug withdrawal. We highlight the unusual appearance of this side effect after a 10-year period of treatment tolerance.

## 2. Clinical Case

**Figure 1:**
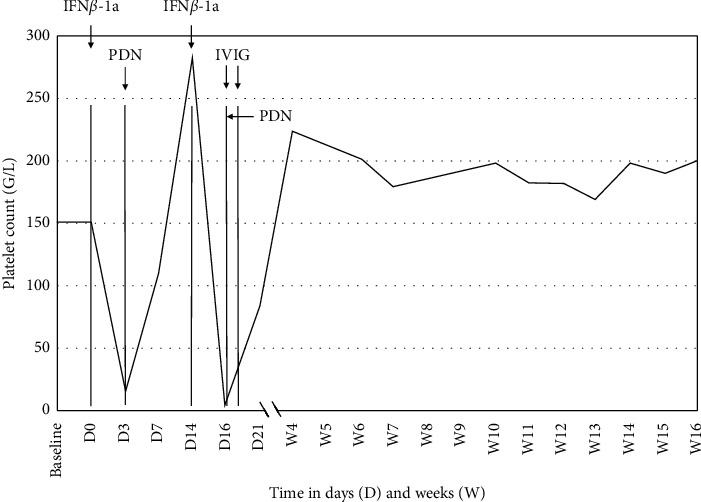
Platelet count at baseline (2 months before), since the last interferon *β*-1a injection before the occurrence of petechial lesions (IFN*β*-1a, D0) and upon rechallenge (D14), and their evolution with subsequent treatments. Prednisone (PDN) is only shown when 1 mg/kg has been initiated. Two administrations of intravenous immunoglobulins (IVIG, 2 g/kg total dose) were performed.

A 52-year-old woman presented to the emergency department with unexplained bruises, petechial lesions on the four limbs and in the mouth, and hematochezia. She was diagnosed with RRMS at age 32 and had been treated for the last ten years with IFN*β*-1a (30 *µ*g) once weekly, without any relapse over the previous 6 years. The last IFN*β*-1a injection was three days before the onset of symptoms (Figure 1, D0, platelet count 152 G/L). Except for a low body mass index (BMI 18 kg/m^2^), no other comorbidities were known and she did not take any medication but clonazepam, calcium, and vitamin D supplementation. Physical examination was otherwise normal, notably regarding fever. Blood examination showed severe thrombocytopenia (13 G/L; reference range: 150–350 G/L). Other blood parameters were normal and included blood smear, coagulation tests, hemolysis parameters, liver and renal functions, proteinuria and urinary sediment, HIV/viral hepatitis B and C/CMV/EBV/*H. pylori* serologies, serum immunofixation, autoantibodies (antinuclear, antineutrophil cytoplasmic antibodies, anti-nucleosome, anti-nucleoproteins), direct and indirect antiglobulin tests, and vitamins dosage (B9 and B12). Abdominal echography was normal. We first made a working diagnosis of ITP and the initial treatment consisted of oral tranexamic acid (1 g tid), corticosteroids (prednisone 1 mg/kg/day), and advice to IFN*β*-1a discontinuation. After 24 hours, the platelet count rose to 57 G/L (Figure 1) and progressive platelet normalization allowed the patient to be discharged after six days. Eleven days after symptom onset, prednisone was tapered from 40 mg/d to 30 mg/d and IFN*β*-1a injections resumed. Two days later, the patient noticed hemorrhagic blistering on her lips. Platelet count dropped to 1 G/L, requiring intravenous immunoglobulins (IVIG, 1 g/kg for 2 days) and a new oral corticosteroids at 1 mg/kg/day. *Fundus oculi* examination was normal. Brain magnetic resonance imaging showed no sign of RRMS activity. Platelet kinetic was rapidly favorable and we then made the diagnosis of immune DITP following IFN*β*-1a treatment. Lifelong drug discontinuation was advised to her neurologist. Unfortunately, between 6 and 10 months after, thrombocytopenia relapsed in the context of a viral infection and of unknown origin, suggesting ITP. Given the high immunogenic status of the patient, she is currently considered for rituximab or ocrelizumab treatment, which are both effective in multiple sclerosis and other immune-mediated disorders, such as ITP [9].

## 3. Discussion

We report the first case of severe immune-mediated DITP following long-term IFN*β*-1a treatment. The causal relationship between the drug and thrombocytopenia was confirmed by a positive drug rechallenge [10].

Thrombocytopenia in DITP usually occurs within 5–10 days after initiation of a new drug or within hours of subsequent exposure [10]. In our case, thrombocytopenia occurred 3 days after the last IFN*β*-1a injection and 1–2 days upon rechallenge. DITP is generally severe (<20 G/L) with concomitant bleeding symptoms and signs (petechial lesions, bruising, and epistaxis) and deaths have been reported. The primary treatment for DITP is to discontinue the suspected causative agent, with an expected platelet count rise within 1–10 days. Patients experiencing mucous blistering or life-threatening bleeding may benefit from IVIG therapy, plasmapheresis, or platelet transfusion. Corticosteroids seem inefficient in the treatment of DITP, unless the patient is at high risk for, or is actively bleeding [10]. Co-occurrence of corticosteroid dose decrease and IFN*β*-1a injection before thrombocytopenia relapse prompted us to continue the steroids for three months. We did not test for the presence of specific drug antibodies. Of note, results would not have led to treatment modification since the detection of drug-platelet antibodies lacks validation and standardization among the variety of techniques and drugs involved in DITP [10].

No such antibodies were previously described for IFN*β*-1a [10]. Clonazepam treatment was never discontinued and therefore an unlikely cause of DITP. Patient's low BMI might have had a possible role in a dose-related toxicity, even though IFN*β*-1a had been well tolerated for 10 years.

DITP can be immune-mediated (drug-dependent antibodies) or non-immune-mediated (bone marrow suppression). Major drugs with known evidence of immune-mediated thrombocytopenia and mechanisms of the development of drug-dependent antibodies are listed in Table 1. Non-immune-mediated thrombocytopenia involves direct myelosuppression through impairment of megakaryocyte survival, proliferation, and maturation (e.g., valganciclovir, IFN*α*/*β*, and chemotherapy).

Thrombocytopenia occurs in a wide variety of contexts that are summarized in Table 1 [11]. Thrombocytopenia and cases of ITP in multiple sclerosis have been described, however, at a lower frequency than potentially fatal thrombotic microangiopathies (uremic hemolytic syndrome and thrombotic thrombocytopenic purpura) [7]. Unlike IFN*α*, IFN*β* has never been associated with immune-mediated DITP.

We reported this serious adverse effect of IFN*β*-1a to the *swiss pharmacovigilance* (*Swissmedic*). Criteria used to establish the relationship with drug-induced thrombocytopenic purpura were all met [12]. Indeed, therapy with the candidate drug preceded the thrombocytopenic event and recovery from thrombocytopenia was complete and sustained after discontinuation of the drug. Other causes of thrombocytopenia were ruled out, except for the possible role of the autoimmune status of multiple sclerosis, which manifested months later as ITP. Finally, a positive drug rechallenge was performed, being considered as a diagnostic criterion.

## 4. Conclusion

This case highlights an unknown but potentially lethal side effect of IFN*β*-1a treatment: immune-mediated DITP. Remarkably, immune-mediated DITP occurred years after treatment initiation. Treatment consists of long-term drug withdrawal and IVIG in case of severe thrombocytopenia with bleeding manifestations. Other forms of severe thrombocytopenia associated with anemia and multiorgan failure have been associated with IFN*β*-1a treatment (thrombotic microangiopathies); however, they present and are treated differently. Immune-mediated DITP has also been described with other disease-modifying therapies for multiple sclerosis (fingolimod, natalizumab, and alemtuzumab), highlighting the importance for neurologists of knowing of this category of possible side effects.

## Figures and Tables

**Table 1:** Left panel: differential diagnosis of thrombocytopenia. Middle panel: drugs with drug-platelet antibodies involved in drug-induced thrombocytopenia (Adapted from https://www.ouhsc.edu/platelets). Right panel: mechanisms of immune-mediated drug-induced thrombocytopenia (adapted from 10).

Differential diagnosis of thrombocytopenia	Drugs implicated in drug-induced thrombocytopenia (immune-mediated platelet destruction)	Mechanisms of drug-dependent antibodies
(i) Pseudothrombocytopenia (ii) Medications (immune-mediated platelet destruction and non-immune suppression of platelet production) (iii) Herbal preparation, food (walnut and sesame seeds), and beverages (tonic water and cranberry juice) (iv) Infectious diseases (CMV, EBV, Parvovirus B19, HIV, VHB, VHC, *H. pylori*, *M. tuberculosis*, and *M. pneumoniae*) (v) Vaccines (measles, influenza, and VHB) (vi) Hematological and solid cancers (vii) Hemolysis and DIC (viii) Hypersplenism (ix) Autoimmune diseases (SLE, scleroderma, Sjögren's disease, and MCTD) (x) Vitamin deficiencies (B9 and B12) or copper deficiency (xi) Thrombotic microangiopathy (TTP and HSU) (xii) Iodinated contrast product	(i) NSAIDs (naproxen, diclofenac, and ibuprofen) (ii) Antibiotics (rifampicin, penicillins, trimethoprim-sulfamethoxazole, and vancomycin) (iii) Antiepileptics (carbamazepine, phenytoin, and valproic acid) (iv) Quinine and quinidine (v) H2-receptor antagonists (ranitidine and cimetidine) (vi) Chlorothiazide and hydrochlorthiazide (vii) Chemotherapy (oxaliplatin and gold) (viii) Glycoprotein IIb/IIIa receptor antagonists (tirofiban, abciximab, and eptifibatide) (ix) Interferon *α*	(i) Classic drug-dependent antibodies targeted to neoepitope created by noncovalent binding of the drug to platelet glycoproteins (ii) Hapten-induced antibodies created by covalent binding of drug to platelet proteins (iii) Specific fiban- and Fab-binding antibodies (glycoprotein IIb/IIIa receptor antagonists) (iv) Autoantibody production in response to a given drug (gold and alemtuzumab) (v) Platelet-localizing immune complexes leading to their activation and clearance

Abbreviations: CMV: cytomegalovirus; DIC: disseminated intravascular coagulation; EBV: Epstein–Barr virus; HIV: human immunodeficiency virus; HUS: hemolytic uremic syndrome; MCTD: mixed connective tissue disease; NSAIDs: Nonsteroidal anti-inflammatory drugs; SLE: systemic lupus erythematosus; TTP: thrombotic thrombocytopenic purpura; VHB/VHC: viral hepatitis B/C.

## Data Availability

No data were used to support this study.
